# Supplementary material for: PRO-IP-seq tracks molecular modifications of engaged Pol II complexes at nucleotide resolution
Source: Nat Commun. 2023 Nov 3;14:7039. doi: 10.1038/s41467-023-42715-3 (PMC10624850; doi:10.1038/s41467-023-42715-3)
Supplement: Supplementary file 3 — Reporting Summary [file 41467_2023_42715_MOESM3_ESM.pdf]

nature portfolio

Corresponding author(s): Anniina Vihervaara, John T. Lis

Last updated by author(s): Aug 27, 2023

Reporting Summary

Nature Portfolio wishes to improve the reproducibility of the work that we publish. This form provides structure for consistency and transparency in reporting. For further information on Nature Portfolio policies, see our Editorial Policies and the Editorial Policy Checklist.

Please do not complete any field with "not applicable" or n/a. Refer to the help text for what text to use if an item is not relevant to your study.
For final submission: please carefully check your responses for accuracy; you will not be able to make changes later.

Statistics

For all statistical analyses, confirm that the following items are present in the figure legend, table legend, main text, or Methods section.

n/a	Confirmed	
☐	☒	The exact sample size (n) for each experimental group/condition, given as a discrete number and unit of measurement
☐	☒	A statement on whether measurements were taken from distinct samples or whether the same sample was measured repeatedly
☐	☒	The statistical test(s) used AND whether they are one- or two-sided<br>*Only common tests should be described solely by name; describe more complex techniques in the Methods section.*
☒	☐	A description of all covariates tested
☒	☐	A description of any assumptions or corrections, such as tests of normality and adjustment for multiple comparisons
☐	☒	A full description of the statistical parameters including central tendency (e.g. means) or other basic estimates (e.g. regression coefficient) AND variation (e.g. standard deviation) or associated estimates of uncertainty (e.g. confidence intervals)
☐	☒	For null hypothesis testing, the test statistic (e.g. F, t, r) with confidence intervals, effect sizes, degrees of freedom and P value noted<br>*Give P values as exact values whenever suitable.*
☒	☐	For Bayesian analysis, information on the choice of priors and Markov chain Monte Carlo settings
☒	☐	For hierarchical and complex designs, identification of the appropriate level for tests and full reporting of outcomes
☒	☐	Estimates of effect sizes (e.g. Cohen's d, Pearson's r), indicating how they were calculated

Our web collection on statistics for biologists contains articles on many of the points above.

Software and code

Policy information about availability of computer code

Data collection	The sequencing data generated and used here are available as complete raw datasets and normalized density profiles in GEO (https://www.ncbi.nlm.nih.gov/geo/query/acc.cgi?acc=GSE200269). All the GSE accession codes are provided in the manuscript. The raw Western Blotting images have been deposited to Mendeley and the link and doi provided in the manuscript file (https://data.mendeley.com/datasets/s44mkg6jmb/1).
Data analysis	We have provided data analysis statement, including a link to GitHub (https://github.com/Vihervaara) where the main pipelines used in the study are provided. All computational tools used are listed in the Methods section and also in the metafile in the GEO. A list of computational tools with the used version is provided also here:<br>fastx_toolkit (0.0.14)<br>fastp (0.12.4)<br>fastq_pair (1.0)<br>fastqc (0.12.1)<br>bedtools (2.30.0)<br>bowtie2 (2.4.5)<br>samtools (1.3.1)<br>bedGraphToBigWig (v4)<br>IGV (2.15.1)

For manuscripts utilizing custom algorithms or software that are central to the research but not yet described in published literature, software must be made available to editors and reviewers. We strongly encourage code deposition in a community repository (e.g. GitHub). See the Nature Portfolio guidelines for submitting code & software for further information.

Data

Policy information about availability of data

All manuscripts must include a data availability statement. This statement should provide the following information, where applicable:

- Accession codes, unique identifiers, or web links for publicly available datasets
- A description of any restrictions on data availability
- For clinical datasets or third party data, please ensure that the statement adheres to our policy

Data availability
The complete raw datasets and normalized density profiles generated (GSE200269) and used (GSE89382; GSE60358; GSE106881; GSE159326) in this study can be accessed via GEO (http://www.ncbi.nlm.nih.gov/geo) and ENCODE (https://www.encodeproject.org) databases.

Research involving human participants, their data, or biological material

Policy information about studies with human participants or human data. See also policy information about sex, gender (identity/presentation), and sexual orientation and race, ethnicity and racism.

Reporting on sex and gender	Does not apply.
Reporting on race, ethnicity, or other socially relevant groupings	Does not apply.
Population characteristics	Does not apply.
Recruitment	Does not apply.
Ethics oversight	Does not apply.

Note that full information on the approval of the study protocol must also be provided in the manuscript.

Field-specific reporting

Please select the one below that is the best fit for your research. If you are not sure, read the appropriate sections before making your selection.

☒ Life sciences ☐ Behavioural & social sciences ☐ Ecological, evolutionary & environmental sciences

Life sciences study design

All studies must disclose on these points even when the disclosure is negative.

Sample size	Three replicates was used in this study, both for PRO-IP-seq and Western Blotting experiments.<br>Regarding analyses of gene expression or Pol II regulation, the sample size was deduced as number of genes that were actively transcribed (n=10,635).
Data exclusions	We did not exclude any data or data points.
Replication	Three replicates was used in this study, both for PRO-IP-seq and Western Blotting experiments.
Randomization	No randomisation was conducted in this study.
Blinding	We did not use blinding in data collection or analyses. Instead, in the PRO-IP-seq protocol we developed here, each sample is barcoded as early as possible the samples (in a replicate) combined into one pool: all the subsequent steps of biochemistry are conducted in this pool, ensuring equal handling. (This could be considered blinding since the pooled samples can only be separated computationally, after the libraries have been sequenced.) The barcoding and pooling was explicitly designed to minimize handling differences and enable as high comparability as possible between samples and replicates. In the data-analyses, each sample is run through the exact same computational pipeline and normalized against the same control sample, which brings the signal intensity to the same scale and allows comparison of Pol II densities between samples and replicates.

Reporting for specific materials, systems and methods

We require information from authors about some types of materials, experimental systems and methods used in many studies. Here, indicate whether each material, system or method listed is relevant to your study. If you are not sure if a list item applies to your research, read the appropriate section before selecting a response.

Materials & experimental systems

n/a	Involved in the study	
☐	☒	Antibodies
☐	☒	Eukaryotic cell lines
☒	☐	Palaeontology and archaeology
☒	☐	Animals and other organisms
☒	☐	Clinical data
☒	☐	Dual use research of concern
☒	☐	Plants

Methods

n/a	Involved in the study	
☒	☐	ChIP-seq
☒	☐	Flow cytometry
☒	☐	MRI-based neuroimaging

Antibodies

Antibodies used	All the antibodies and their identification codes are provided in the manuscript. Pol II antibodies target the largets RPB1 subunit.<br><br>N-terminal domain of RPB1 (polyclonal, Santa Cruz, sc899, RRID: AB_632359, 1:200 dilution in WB, 5ug in PRO-IP-seq)<br>Unphosphorylated Pol II CTD (Abcam, 8WG16, RRID: AB_2268549, 5ug in PRO-IP-seq)<br>Serine-2-phosphorylated Pol II CTD (monoclonal, Millipore, 04-1571, 3E10, RRID: AB_2631403, 1:1000 dilution in WB, 5ug in PRO-IP-seq)<br>Serine-2-phosphorylated Pol II CTD (MBL International, Clone MABI0602, RRID: AB_2747403, 5ug in PRO-IP-seq)<br>Serine-5-phosphorylated Pol II CTD (monoclonal, Millipore, 04-1572, 3E8. RRID: AB_2801296, 1:1000 dilution in WB, 5ug in PRO-IP-seq)<br>Serine-5-phosphorylated Pol II CTD (MBL International, Clone MABI0603, RRID: AB_2728736, 5ug in PRO-IP-seq)<br>Serine-7-phosphorylated Pol II CTD (monoclonal, Millipore 04-1570, 4E12, RRID: AB_2801298, 1:1000 dilution in WB, 5ug in PRO-IP-seq).<br>β-tubulin (Abcam, ab6046, RRID: AB_2210370, 1:5000 dilution in WB)<br>Negative IgG control antibody (Santa Cruz, sc-2027, RRID: AB_737197, 5ug in PRO-IP-seq).
Validation	We verified that the antibodies produced the expected size pattern with Western Blotting. The specificity of the antibodies for the indicated Pol II CTD modifications has been characterized in detail by Chapman et al., 2007, Science.<br>[Chapman, R.D., Heidemann, M., Albert, T.K., Mailhammer, R., Flatley, A., Meisterernst, M., Kremmer, E. and Eick, D. (2007). Transcribing RNA polymerase II is phosphorylated at CTD residue serine-7. Science 318, 1780-1782.]<br>Additionally, every antibody was chosen after careful literature search, including ensuring the manufacturer provided verification of the antibody.

Eukaryotic cell lines

Policy information about cell lines and Sex and Gender in Research

Cell line source(s)	Human K562 erythroleukemia cells, originating from ATCC and obtained from Prof. Lea Sistonen laboratory, Åbo Akademi University, Turku, Finland. We have previously used and characterized this exactly same K562 cell line in publications: Vihervaara et al., 2017, Nature Communications and Vihervaara et al., 2021, Molecular Cell.<br>The spike-in wild-type male MEFs originated from Prof. Susan Lindquist laboratory, MIT, Boston, USA. These cells have previously been verified and used by us in publications: Mahat et al., 2016, Molecular Cell and Vihervaara et al., 2021 Molecular Cell.
Authentication	K562 and MEF cells used in this study display the characteristic morphology, cell cycle, proliferation and gene expression program as expected from previous studies by us and others. Both MEFs and K562 cells have been analyzed with microscopy (wide-field live microscopy) in optimal growth conditions (37°C) during a heat shock response (42°C monitored for 6 hours). K562 cells have additionally been analyzed for their cell cycle progression using FACS and counting of cells to quantify proliferation.
Mycoplasma contamination	The cells were tested for mycoplasma prior to initiating the study. No indication for mycoplasma was detected.
Commonly misidentified lines<br>(See ICLAC register)	We did not use any commonly misidentified cell lines.

This checklist template is licensed under a Creative Commons Attribution 4.0 International License, which permits use, sharing, adaptation, distribution and reproduction in any medium or format, as long as you give appropriate credit to the original author(s) and the source, provide a link to the Creative Commons license, and indicate if changes were made. The images or other third party material in this article are included in the article's Creative Commons license, unless indicated otherwise in a credit line to the material. If material is not included in the article's Creative Commons license and your intended use is not permitted by statutory regulation or exceeds the permitted use, you will need to obtain permission directly from the copyright holder. To view a copy of this license, visit http://creativecommons.org/licenses/by/4.0/
