# Supplementary material for: PRO-IP-seq tracks molecular modifications of engaged Pol II complexes at nucleotide resolution
Source: Nat Commun. 2023 Nov 3;14:7039. doi: 10.1038/s41467-023-42715-3 (PMC10624850; doi:10.1038/s41467-023-42715-3)
Supplement: Supplementary file 4 — Source Data [file 41467_2023_42715_MOESM4_ESM.zip › Vihervaara_SourceDataFiles_Sept2023.pdf]

Source data 1

Shows Western Blotting films and indicates which part of them (dashed red box) is included in Fig. 5e.

Pol II antibodies shown in Fig 5e

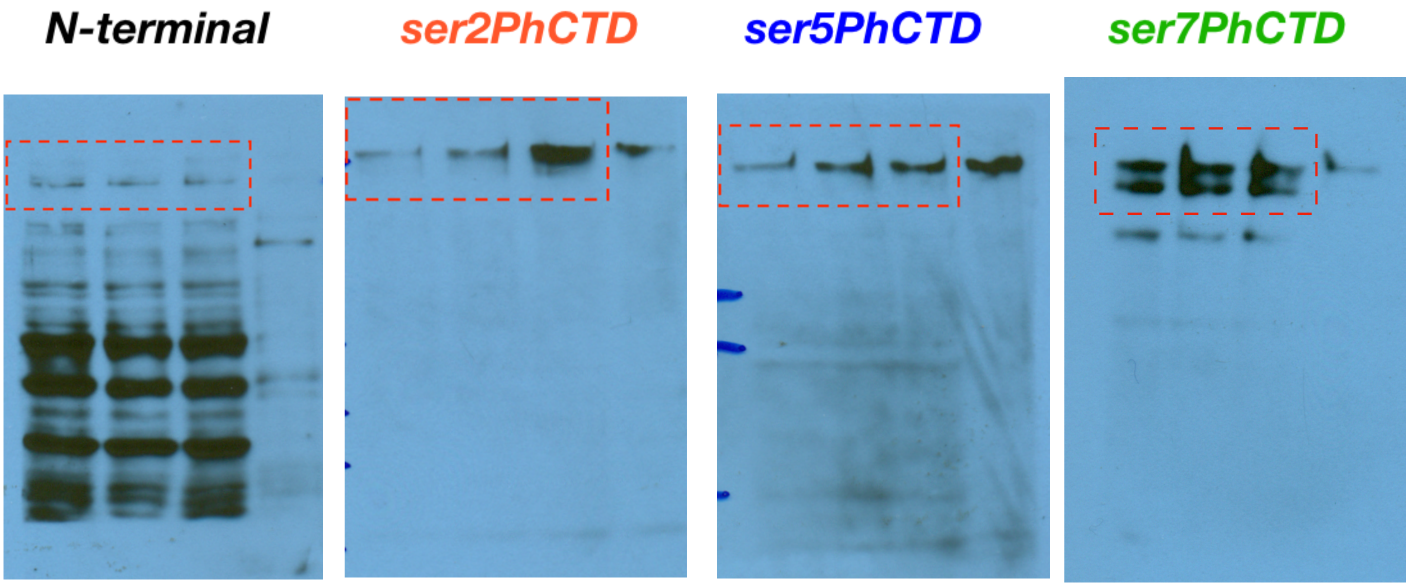

beta-tubulin (loading control) shown in Fig 5e

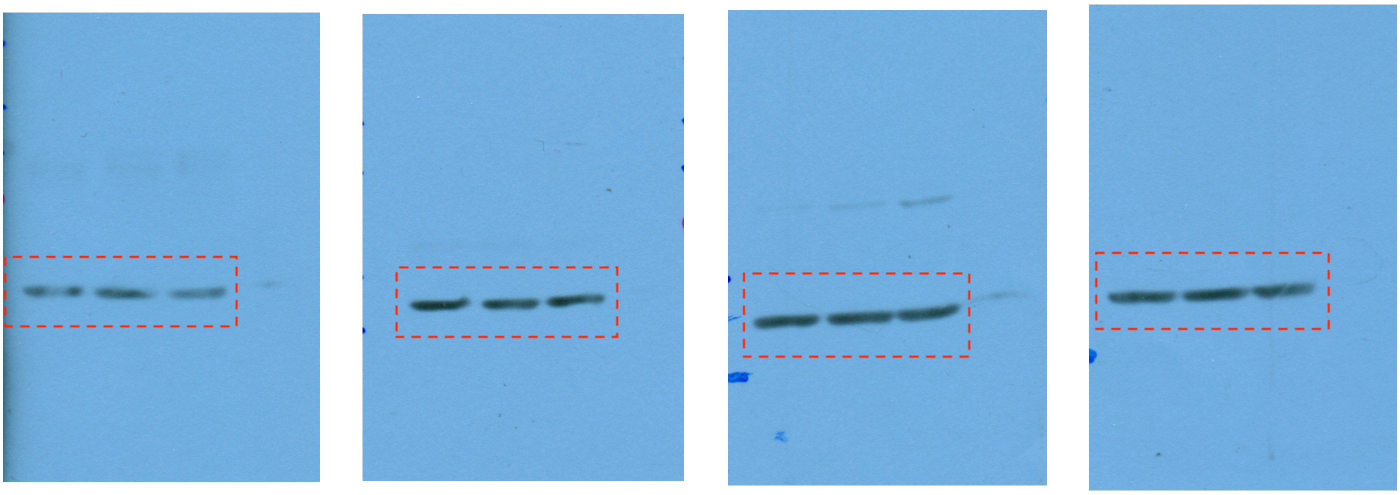

For positions of the protein markers, please see source data 2 (Pol II antibodies) and Source Data 3 (tubulin antibodies)

Source data 2

Uncropped Western Blotting films from the same experiment as shown in Source Data 1. In this version, the positions of size markers are clearly visible for the Pol II antibodies. Please note that the exposure time might differ from the data provided in Source Data 1.

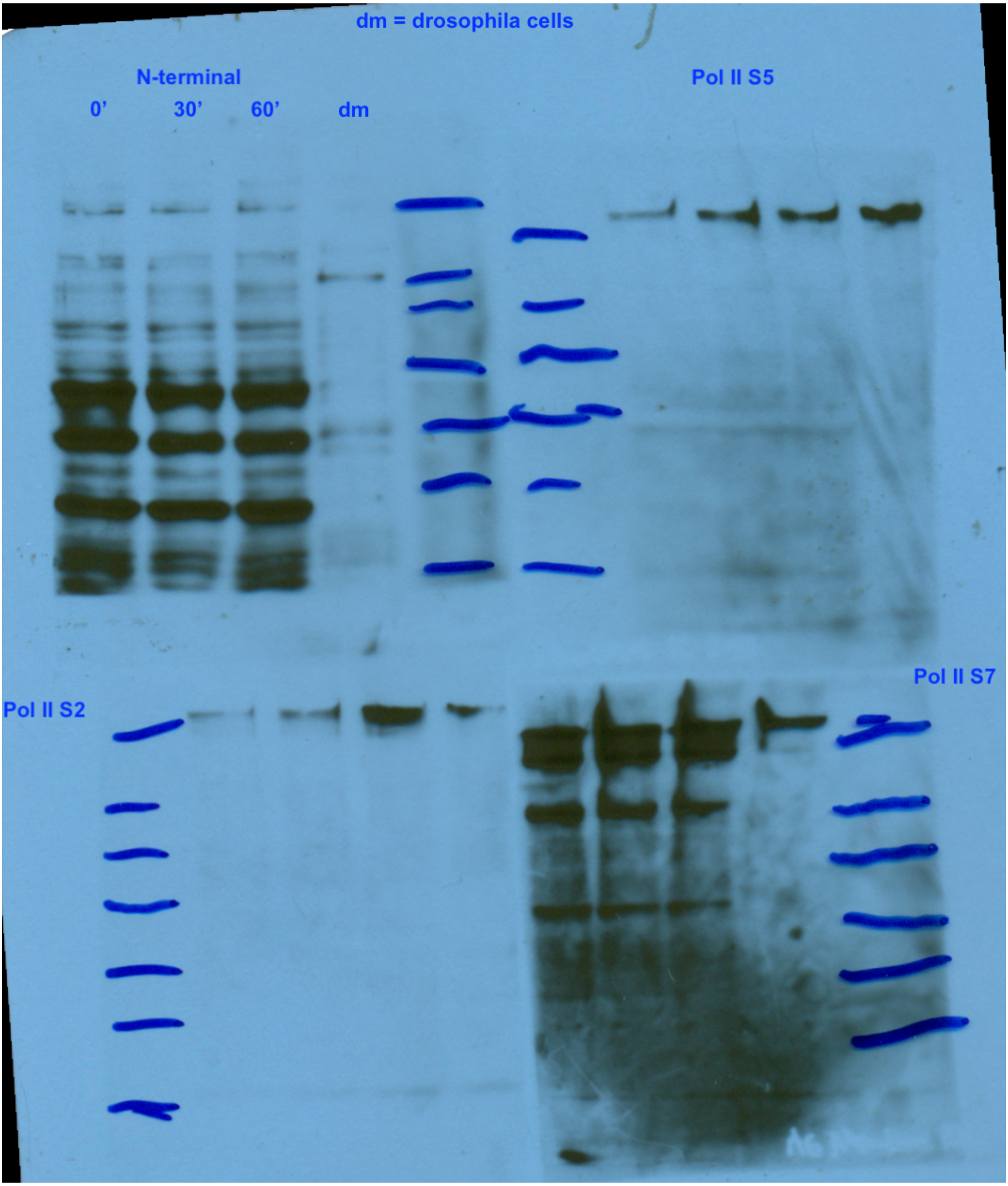

Source data 3

Uncropped Western Blotting films from the same experiment as shown in Source Data 1. In this version, the positions of protein size markers are visible. Please note that in the leftmost membrane the protein markers are faintly indicated against the dark background at left. In the tubulin control for Ser7PhCTD (rightmost membrane), the protein marker was loaded to right of the ‘dm’ lane but marked here to the left.

beta-tubulin (loading control) shown in Fig 5e, including the positions of protein marker bands.

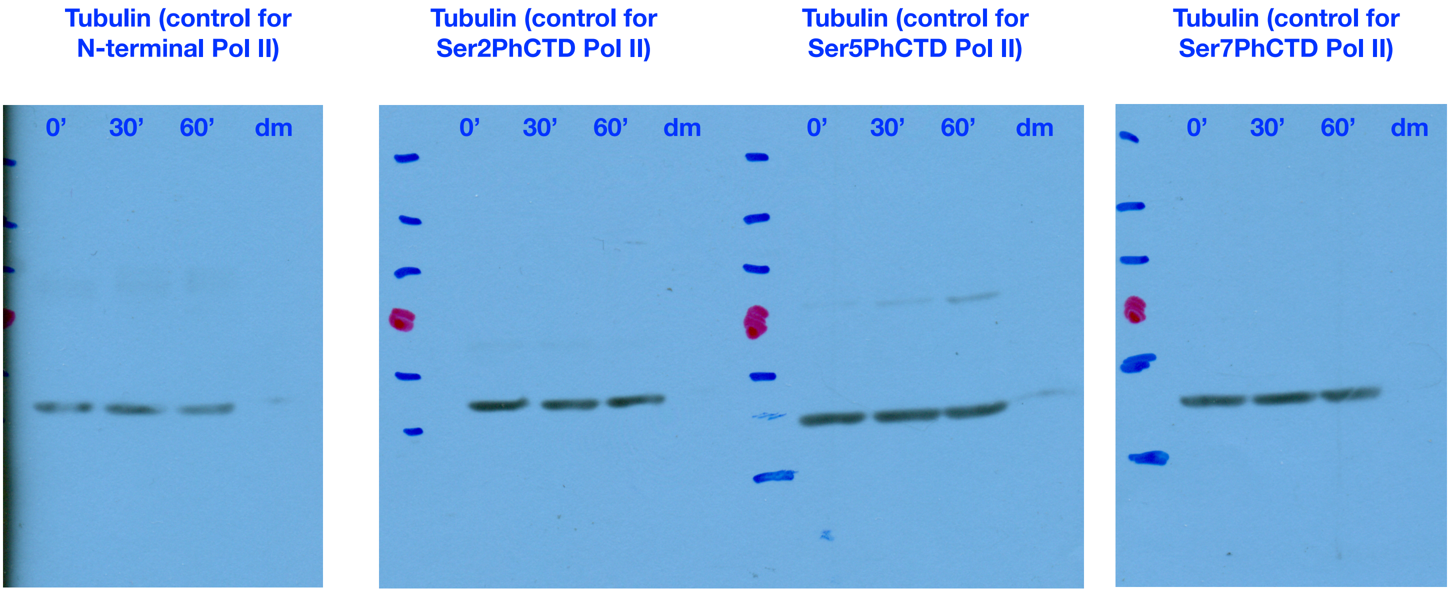

dm = Drosophila cells run in the same experiment but not shown in this study

Source data 4

Raw data (signal intestines) for quantification of WB shown in Fig 5f

Replicate	Pol II antibody	Timepoint (min)	Pol II intensity	Tubulin intensity	Normalized Pol II value
Rep1	N-term	0	9394,267	10518,61	0,893109166
Rep1	N-term	30	7850,196	12354,903	0,635391148
Rep1	N-term	60	6272,368	8191,296	0,765735727
Rep1	ser2Ph	0	2083,74	12037,711	0,173101016
Rep1	ser2Ph	30	5013,368	10066,468	0,498026517
Rep1	ser2Ph	60	21457,794	10059,711	2,133042788
Rep1	ser5Ph	0	4721,004	13782,196	0,34254367
Rep1	ser5Ph	30	11826,48	13818,903	0,855819018
Rep1	ser5Ph	60	10816,045	12521,146	0,863822289
Rep1	ser7Ph	0	25901,957	12498,075	2,072475721
Rep1	ser7Ph	30	33936,898	13522,61	2,509641112
Rep1	ser7Ph	60	26619,099	11086,439	2,401050418
Rep2	N-term	0	11287,652	14775,974	0,763919319
Rep2	N-term	30	23965,501	25101,066	0,954760288
Rep2	N-term	60	17387,945	29524,652	0,588929719
Rep2	ser2Ph	0	5097,125	17938,439	0,284145404
Rep2	ser2Ph	30	6374,823	17950,53	0,355132857
Rep2	ser2Ph	60	14520,108	16262,56	0,892854999
Rep2	ser5Ph	0	4570,066	17992,974	0,253991697
Rep2	ser5Ph	30	12368,217	18601,631	0,664899599
Rep2	ser5Ph	60	10874,267	18212,359	0,59708174
Rep2	ser7Ph	0	4573,326	13742,409	0,332789251
Rep2	ser7Ph	30	5940,054	15743,844	0,377293754
Rep2	ser7Ph	60	2936,669	12899,258	0,227661855
Rep3	N-term	0	3770,569	20451,309	0,184368101
Rep3	N-term	30	6513,983	18773,723	0,346973427
Rep3	N-term	60	6660,832	16695,752	0,398953698
Rep3	ser2Ph	0	8634,451	19707,773	0,438124135
Rep3	ser2Ph	30	9914,472	19255,652	0,514886331
Rep3	ser2Ph	60	10347,279	20375,48	0,507829951
Rep3	ser5Ph	0	5668,279	21712,685	0,261058409
Rep3	ser5Ph	30	23466,676	27826,685	0,843315544
Rep3	ser5Ph	60	23414,019	28474,371	0,822283976
Rep3	ser7Ph	0	15182,572	13834,53	1,09744039
Rep3	ser7Ph	30	19780,836	16531,51	1,196553491
Rep3	ser7Ph	60	18982,421	18099,995	1,048752831
